# Case report: *Mycobacterium chimaera*-induced lymph node infection in a patient with chronic myeloproliferative neoplasm misdiagnosed as tuberculous lymphadenitis

**DOI:** 10.3389/fpubh.2024.1387722

**Published:** 2024-09-24

**Authors:** Yingqian Sun, Chengliang Zhang, Bin Lu, Jun Chen, Xinling Pan

**Affiliations:** ^1^Clinical Laboratory, Affiliated Dongyang Hospital of Wenzhou Medical University, Dongyang, Zhejiang, China; ^2^Department of Infectious Diseases, Affiliated Dongyang Hospital of Wenzhou Medical University, Dongyang, Zhejiang, China; ^3^Department of Medical Imaging, Affiliated Dongyang Hospital of Wenzhou Medical University, Dongyang, Zhejiang, China; ^4^Department of Biomedical Sciences Laboratory, Affiliated Dongyang Hospital of Wenzhou Medical University, Dongyang, Zhejiang, China

**Keywords:** non-tuberculous mycobacteria, *Mycobacterium avium* complex, *Mycobacterium chimaera*, lymphadenitis, diagnosis, case report

## Abstract

Herein, we report a case of lymphadenitis caused by *Mycobacterium chimaera*. A 54-year-old woman with chronic myeloproliferative neoplasm was admitted to the hospital with cervical lymphadenopathy. After preliminary exclusion of various diseases such as lymphoma, Epstein–Barr virus infection, and autoimmune disease, a lymph node biopsy specimen showed epithelioid granulomatous lymphadenitis with caseous necrosis, epithelial-like cells, and multinucleated giant cells as seen in tuberculosis (TB). Although *Mycobacterium tuberculosis* was never isolated, diagnostic anti-TB treatment was commenced. Following over 9 months of treatment, there was no significant reduction in the size of her cervical lymph nodes, and she continued to experience recurrent low-grade fevers. One sample from the fourth lymph node biopsy tested negative for metagenomic next-generation sequencing (mNGS), and another sample tested positive in the BACTEC MGIT960 liquid culture system, identifying the strains as *Mycobacterium chimaera*. Anti-non-tuberculous mycobacteria (NTM) therapy was initiated, and the patient achieved symptom improvement. In conclusion, NTM lymphoid infection is easily misdiagnosed as long-term etiologic negativity.

## 1 Introduction

Non-tuberculous mycobacteria (NTM) refer to mycobacteria other than *Mycobacterium leprae* and *Mycobacterium tuberculosis* complex. Over the past few decades, NTM epidemics have increased globally (1–3). As NTM closely resembles *Mycobacterium tuberculosis* in pathogenesis, clinical manifestations, and pathology, NTM infection, particularly extrapulmonary NTM infection, tends to be atypical and highly uncommon. Consequently, its diagnosis is often challenging and delayed (4, 5). *Mycobacterium avium* complex is a major NTM associated with infections (3), and *Mycobacterium chimaera* (MC) is a member of the *Mycobacterium avium* complex. Lymphadenitis caused by *Mycobacterium avium* complex or another NTM is more common in children than in adults (6), and extrapulmonary NTM infections in adults usually occur in individuals with congenital or acquired immunodeficiency (7). Herein, we present a case of lymph node infection caused by MC. Four lymph node biopsies and extensive laboratory, imaging, and pathological examinations were performed. MC was obtained by lymph node culture, and symptom improvement was achieved. The entire process of diagnosis and treatment may provide a reference for the clinical diagnosis and treatment of similar patients. This case study can improve clinicians’ understanding of NTM-related diseases, reduce misdiagnoses and missed diagnoses, and avoid delays in treatment.

## 2 Case description

On 31 March 2022, a 54-year-old woman was admitted to our hospital after finding a mass in the right cervical root 4 days prior to admission. She had a 6-year history of thrombocytosis and had no other relevant medical or surgical history. Physical examination revealed a hard, fixed mass approximately 3 cm in diameter in the right cervical lymph node, with indistinct boundaries and no tenderness. The patient did not exhibit symptoms such as chills, fever, cough, expectoration, weight loss, or night sweats. To elucidate the nature of the lymphadenopathy, the following tests were conducted.

### 2.1 Laboratory tests

Blood cytology showed an elevated platelet count (825 × 10^9^/L), white blood cell count within normal range (7.06 × 10^9^/L), reduced absolute Lymphocytes count (821 × 10^6^/L), and reduced differential lymphocyte count (T Lymphocytes: 799 × 10^6^/L, CD3^+^CD4^+^ T cells: 456 × 10^6^/L, CD3^+^CD8^+^ T cells: 308 × 10^6^/L, B Lymphocytes: 9 × 10^6^/L, and NK cells: 8 × 10^6^/L). An Epstein-Barr virus (EBV) test revealed evidence of a previous infection with 6.77 × 10^2^ DNA copies/ml of a whole blood sample (↑400), EBV capsid antigen IgG antibody titer >750 U/mL, and EBV nuclear antigen IgG antibody titer >600 U/mL; EBV capsid antigen IgM antibody was normal. Moreover, neurogenic specific enolase was 25.48 ng/mL (↑ < 16.3), cytokeratin 19 was 5.85 ng/mL (↑ < 3.3), and uric acid was 495 μmol/L (↑155–357). Anti-nuclear antibody was positive at 1:100 nuclear granule of the main karyotype, and rheumatoid factor was 26 IU/mL (↑ < 20), while tests for anti-Streptolysin “O,” anti-cyclic citrullinated peptide antibody, anti-neutrophil cytoplasmic antibody, immunoglobulin G4, and anticardiolipin antibody were all negative. The T-SPOT test was non-reactive. Serological tests for erythrocyte sedimentation rate, C-reactive protein, procalcitonin, human immunodeficiency virus, hepatitis B, hepatitis C, and syphilis tests were normal. Additionally, her immunoglobulin A, M, G, complement C3, C4, interleukin-2 (IL-2), IL-4, IL-6, IL-10, interferon-*γ*, and tumor necrosis factor levels were within the normal range. Laboratory parameters on admission are presented in Table 1. Abnormal myeloid precursor cells were detected in her bone marrow morphology and flow cytometry, suggesting a link with myeloproliferative neoplasm. According to the result of genetic testing, mutations in ASXL1, SETBP1, U2AF1, and PTPN11 were observed, and a diagnosis of chronic myeloproliferative neoplasm-primary thrombocytosis was made. Hydroxyurea and aspirin were administered as anticoagulants and for the treatment of thrombocytosis.

**Table 1:** Patient’s laboratory parameters on first and second admissions to Affiliated Dongyang Hospital of Wenzhou Medical University.

Parameter	Value	Reference range
First admission		
White blood cell count [10^9^/L]	7.06	3.5–9.5
Lymphocytes [10^6^/L]	821↓	1,459–2,633
T Lymphocytes (CD3^+^) [10^6^/L]	799↓	919–1817
CD3^+^CD4^+^ T cells [10^6^/L]	456↓	467–949
CD3^+^CD8^+^ T cells [10^6^/L]	308	292–830
B Lymphocytes [10^6^/L]	9↓	107–319
NK cells [10^6^/L]	8↓	162–590
Platelets [10^9^/L]	825↑	125–350
Epstein–Barr virus DNA [copies/ml]	677↑	400
Epstein–Barr virus capsid antigen IgG antibody[U/ml]	>750↑	<20
Epstein–Barr virus nuclear antigen IgG antibody [U/ml]	>600↑	<20
Epstein–Barr virus capsid antigen IgM antibody [U/ml]	0.78	<40
Procalcitonin [ng/ml]	0.085	<0.1
C-reactive protein (serum) [mg/L]	3.4	<5
Serological tests for erythrocyte sedimentation rate [mm/h]	29	<38
Neurogenic-specific enolase [ng/ml]	25.48↑	<16.3
Cytokeratin 19 [ng/ml]	5.85↑	<3.3
IgG4 [g/L]	0.82	<2.01
IgG [g/L]	14.8	7–16
IgA [g/L]	1.80	0.70–4.00
IgM [g/L]	0.62	0.40–2.30
Complement C3[g/L]	1.14	0.80–1.60
Complement C4[g/L]	0.18	0.10–0.50
IL-2 [pg/ml]	2.58	<7.5
IL-4 [pg/ml]	<2.44	<8.56
IL-6 [pg/ml]	4.01	<5.4
IL-10 [pg/ml]	2.98	<12.9
Interferon-γ [pg/ml]	18.25	<23.1
Tumor necrosis factor [pg/ml]	<2.44	<16.5
Uric acid [μmol/l]	495↑	155–357
Rheumatoid factor [IU/ml]	26↑	<20
Anti-streptolysin “O” [IU/ml]	<20	<160
Anti-cyclic citrullinated peptide antibody [IU/ml]	<8	<17
Anticardiolipin antibody	Neg.	
Anti-nuclear antibody	Positive at 1:100 nuclear granule of the main karyotype	
Anti-neutrophil cytoplasmic antibody	Neg.	
Hepatitis B surface antigen	Neg.	
Hepatitis B surface antibody	Neg.	
Hepatitis B e antigen	Neg.	
Hepatitis B e antibody	Neg.	
Hepatitis B core antibody	Neg.	
*Treponema pallidum* antibody	Neg.	
Human immunodeficiency virus antibody/p24 antigen	Neg.	
Hepatitis C virus antibody	Neg.	
T-SPOT	Neg.	
Second admission		
White blood cell count [10^9^/L]	7.64	3.5–9.5
Lymphocytes [10^6^/L]	788↓	1,459–2,633
T Lymphocytes (CD3^+^) [10^6^/L]	739↓	919–1817
CD3^+^CD4^+^ T cells [10^6^/L]	405↓	467–949
CD3^+^CD8^+^ T cells [10^6^/L]	300	292–830
B Lymphocytes [10^6^/L]	6↓	107–319
NK cells [10^6^/L]	27↓	162–590
Platelets [10^9^/L]	560↑	125–350
Epstein–Barr virus DNA [copies/ml]	1930↑	400
Epstein–Barr virus capsid antigen IgG antibody [U/ml]	>750↑	<20
Epstein–Barr virus nuclear antigen IgG antibody [U/ml]	>600↑	<20
Epstein–Barr virus capsid antigen IgM antibody [U/ml]	0.77	<40
Uric acid [μmol/l]	495↑	155–357
rheumatoid factor [IU/ml]	24↑	<20
Anti-nuclear antibody	Neg.	
T-SPOT	Neg.	

IL, interleukin; Neg., negative.

### 2.2 Medical imaging

**Figure 1:**
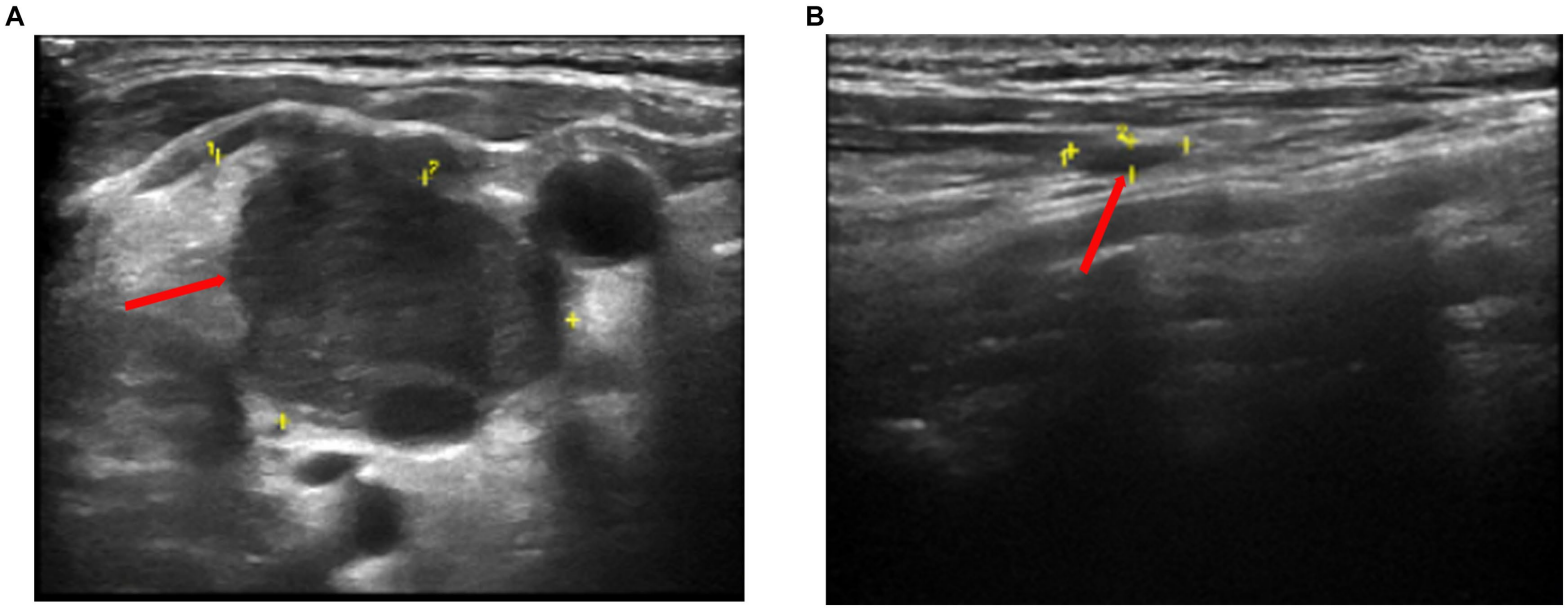
Color doppler sonography showed multiple echogenic lymph nodes of different sizes on both clavicles. **(A)** The larger one on the right is approximately 28 mm × 20 mm in size (yellow section and red arrow). **(B)** The larger one on the left is approximately 25 mm × 8 mm in size (yellow section and red arrow).

**Figure 2:**
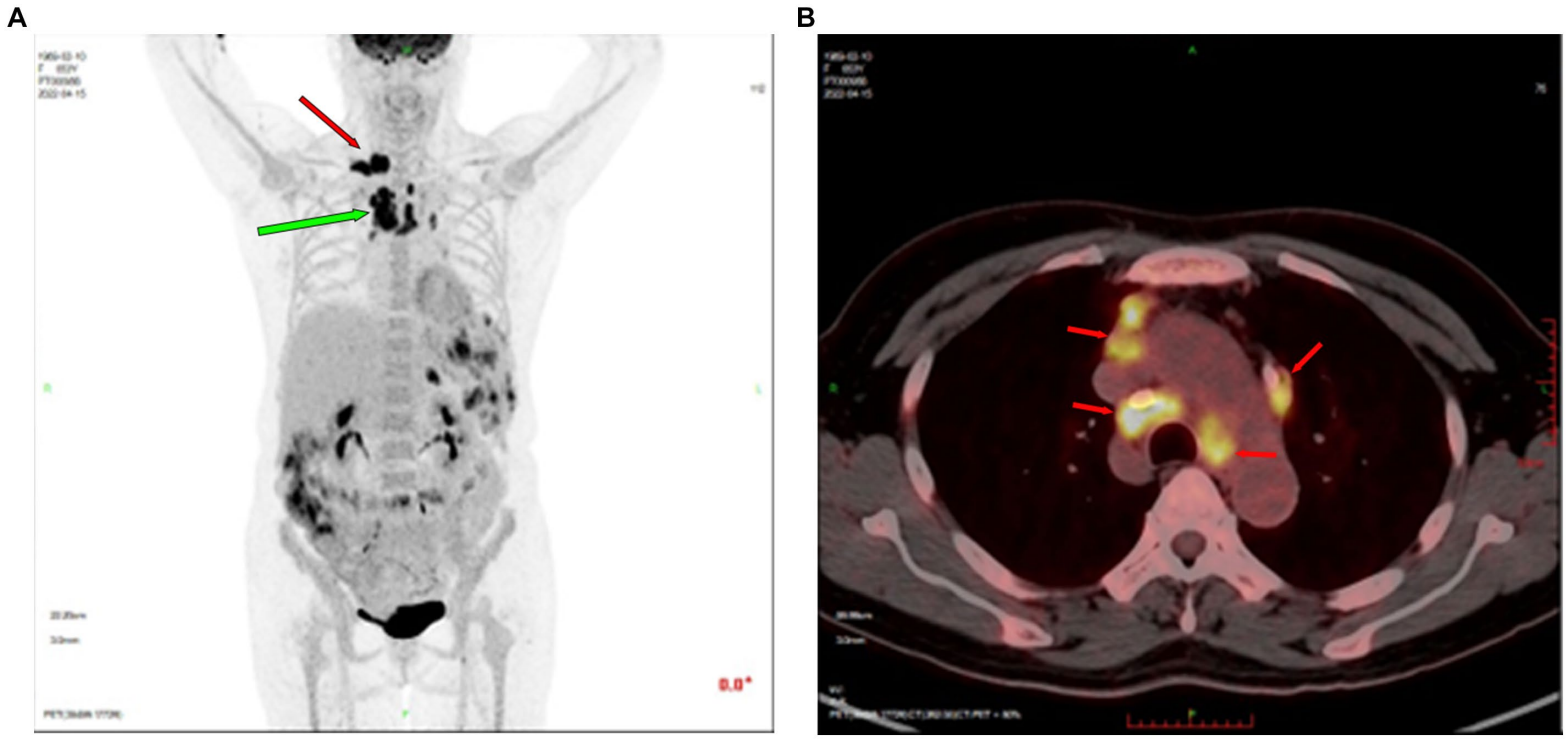
Positron emission tomography-CT revealed excessive FDG accumulation in multiple lymph nodes. **(A)** The right supraclavicular lymph node, approximately 27 mm in diameter, showed increased FDG uptake (SUVmax = 15.6, indicated by red arrow). Enlarged mediastinal lymph nodes showed increased FDG uptake (green arrow). **(B)** Excessive FDG accumulation in mediastinal lymph nodes (red arrow). CT, computed tomography; FDG, fluorodeoxyglucose; SUVmax, maximum standard uptake value.

Color Doppler ultrasound revealed multiple lymphadenopathies in the bilateral supraclavicular (Figures 1A,B), neck, armpit, and inguinal regions. Plain chest computed tomography (CT) and X-ray images showed clear pulmonary textures in both lungs, and there were no exudates or space-occupying lesions in the lung parenchyma. Enhanced CT revealed scattered infections in both lungs, accompanied by a pleural reaction on both sides. Multiple enlarged lymph nodes were observed in the neck and mediastinum, along with an enlarged spleen and multiple low-density foci within. A cyst was noted in the right kidney, and a small amount of fluid was present in the pelvic cavity. Her whole-body positron emission tomography-CT (PET-CT) examination revealed excessive fluorodeoxyglucose (FDG) accumulation in multiple swollen lymph nodes (bilateral supraclavicular, neck, mediastinum, and left inguinal regions), particularly in the right supraclavicular region, measuring approximately 27 mm in diameter, with increased 18-FDG uptake, having a maximum standard uptake value (SUVmax) of 15.6 (Figure 2A). Excessive accumulation was noted in the mediastinal lymph nodes, with an SUVmax of 14.0. The largest node measured approximately 25 × 13 mm (Figures 2A,B). Additionally, multiple circular low-density foci of varying sizes were observed in the spleen, the largest of which was approximately 30 mm in diameter, with an elevated FDG uptake and SUVmax of 10.8. There was also diffuse FDG uptake in the bone marrow of the axial skeleton and limbs, with an SUVmax of 4.3. Considering the patient’s history and atypical symptoms of TB, the likelihood of lymphoma was considered to be high.

### 2.3 Pathological examination

**Figure 3:**
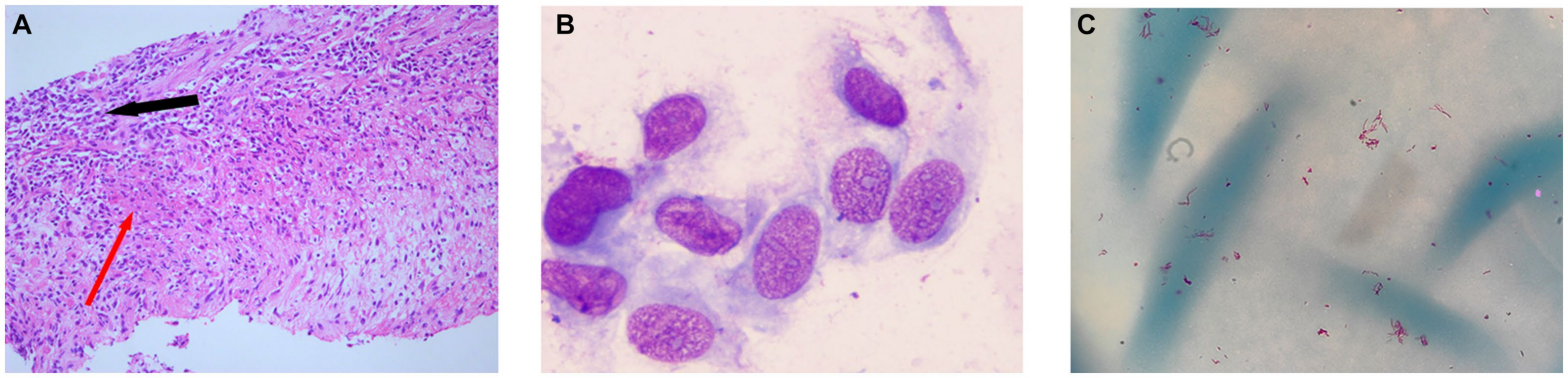
**(A)** Lymph node biopsy: granulomatous inflammation (red arrow) and acute and chronic inflammatory cell infiltration (black arrow). **(B)** Epithelial-like cells by Wright–Giemsa stain. **(C)** Acid-fast bacteria (red color) were isolated from the MGIT960 liquid culture tube after centrifugation concentration and acid-fast staining.

Two lymph node biopsies were performed during the hospital stay, revealing chronic lymphadenitis with local granuloma formation (Figure 3A), focal necrosis, and acute and chronic inflammatory cell infiltration (Figure 3A). Epithelial-like cells (Figure 3B) and multinucleated giant cells were observed locally, and lymphoid structures were partially absent. Lymphocytes had either a mass or diffuse distribution. Neutrophils and plasma cells were also observed, and there was evidence of proliferation of fibrous tissue. The results of immunohistochemical staining were as follows: acid-fast staining (−), periodic acid-Schiff staining (−), Alcian blue-periodic acid-Shiff (−), S-100 (−), CD3 (t-cell +), Cd5 (t-cell +), CD20 (b-cell +), CD79a (B-cell +), CD1a (−), CD68 (histiocytes +), and Myeloperoxidase (neutrophil +). No malignant cells were found in the liquid-based cytology of the left supraclavicular lymph node. At the same time, flow cytometry of the biopsies showed no lymphoma cells.

Before further examination to confirm the diagnosis, the patient voluntarily transferred to the First Affiliated Hospital of Zhejiang University. On 13 May 2022, biopsies were performed on the left inguinal and right cervical lymph nodes. The report showed negative results for the right cervical lymph nodes: Xpert MTB/RIF assay (Cepheid), periodic acid-Schiff staining, and fungus (Fluorescence *in situ* hybridization). Histopathological analysis of the right cervical lymph node revealed granulomatous inflammation with focal caseous necrosis. The left inguinal lymph nodes exhibited reactive hyperplasia and were treated with diagnostic anti-TB treatment for lack of etiological evidence. The patient commenced treatment with isoniazid, rifampicin, ethambutol, and pyrazinamide on 15 May 2022. On 15 July 2022, CT scans of the chest and abdomen revealed multiple enlarged lymph nodes in the right clavicle and mediastinum post-anti-TB treatment, indicating a reduction in size compared to that of previous scans. Infection was observed in both lungs, showing signs of absorption and scattered proliferative foci. On 28 July 2022, isoniazid, rifampicin, and ethambutol were continued as anti-TB treatments.

On 31 January 2023, she was admitted to our hospital for a second time because of dizziness and chest tightness for 3 days. Despite undergoing more than 9 months of anti-TB therapy, the patient showed little improvement, and there were recurring symptoms accompanied by a low-grade fever. During hospitalization, the purified protein derivative test was strongly positive, and the T-SPOT was negative. Blood cytology showed a decrease in platelet count when compared to the first admission (560 × 10^9^/L), while the absolute counts of total lymphocytes and multiple subpopulations were still low (Table 1). EBV were 1930 DNA copies/ml of a whole blood sample and EBV capsid antigen IgM antibody was normal. Anti-nuclear antibody was negative (Table 1). To confirm the cause of lymphadenopathy, a subsequent biopsy was performed. The Xpert MTB/RIF assay (Cepheid) yielded a negative result on the left supraclavicular lymph node biopsy, and metagenomic next-generation sequencing (mNGS) did not detect any pathogenic microorganisms in the tissues. Another sample was inoculated in MGIT960 for 11 days, where acid-fast bacteria were detected (Figure 3C). The strain was identified as MC by sequencing heat shock proteins 65 (8) on 21 February 2023. Following a positive result in the tube, the tissue initially sent for mNGS was also inoculated in MGIT960 but remained negative after 6 weeks. We inferred the presence of an NTM infection based on (1) low-grade fever, (2) high uptake of PET-CT in patients with inflammation and NTM infection, (3) histopathological examination, and (4) positive culture results of MGIT960. As the patient had been discharged from the hospital, we called her with the news that MC had been detected and suggested that she come to the hospital for treatment on 25 March 2023.

For personal reasons, she postponed returning to our hospital until 7 May 2023. At this point, we initiated anti-NTM treatment with azithromycin, linezolid, rifampicin, and moxifloxacin, and her condition improved. After discharge from the hospital, she stopped the medicines on 6 June 2023, owing to physical intolerance. On 8 August 2023, she was hospitalized again for pain and swelling of the right cervical lymph nodes and was treated with daily moxifloxacin, rifampicin, azithromycin, and doxycycline. At her follow-up after more than 6 months of taking these medications, no tenderness was observed in the right cervical lymph nodes, the swelling had decreased, and the absolute lymphocyte count (760 × 10^6^/L) was still below the lower limit of normal, platelet count was 380 × 10^9^/L. After 11 months of continuous medication, an enhanced CT scan was performed on 10 July 2024, which revealed that the low-density lesions in the spleen were fewer and smaller than previously observed, with the largest lesion measuring approximately 7 mm in its longest dimension. Color Doppler ultrasound revealed that the cervical lymph nodes were much smaller than before, showing that the treatment had been effective.

## 3 Discussion

Lymphadenopathy is a common clinical symptom caused by the proliferation of cells in the lymph nodes or infiltration of tumor cells. Notably, it can occur in several diseases. Our patient’s lymphadenopathy involved multiple parts of the body. EBV is closely associated with some types of lymphoma. Our patient’s EBV DNA levels were slightly elevated, and the splenic changes along with abnormally high uptake on PET-CT raised suspicions of lymphoma. Moreover, the atypical nature of the infection initially posed challenges for differential diagnosis. Furthermore, as the patient’s swollen lymph nodes were located near blood vessels and nerves, the tissue samples could only be obtained through fine needle aspiration, which is inherently limited. Moreover, the detection of positive bacteria in only one tube of liquid medium suggests that low bacterial counts may also present challenges for laboratory testing. It is recommended that patients who are unable to undergo a surgical excision biopsy should be considered for multipoint puncture with a core needle. In conjunction with mNGS, various molecular biology techniques, as well as solid and liquid TB cultures, can improve the detection rate (9–11). Furthermore, some studies indicate that in cases where NTM disease is suspected, avoidance of certain antibiotics during specimen collection is advised, specifically macrolides, quinolones, aminoglycosides, sulfamethoxazole-trimethoprim, and linezolid. Ideally, samples should be collected at least 2 weeks after the discontinuation of these medications, if necessary (12, 13). This is difficult to perform clinically; however, in patients with mild disease, as many specimens as possible are collected after discontinuation to increase the detection sensitivity of NTM.

The NTM widely exists in water, soil, dust, and other natural environments. Planting soil, hospital water, and even shower heads can be rich in *Mycobacterium avium* complex. Human exposure to NTM is common, but epidemics of NTM infection are fairly infrequent, suggesting that NTM has low-to-moderate pathogenicity and that host risk factors play an integral role in vulnerability to NTM disease (14). Individuals with immunodeficiencies or underlying diseases are susceptible to NTM infection. The patient is a menopausal female, living in the city and not involved in farm work. She had no history of acupuncture, surgery, or use of immunosuppressants. We suggested that the ongoing reduction in the counts of T, B, and natural killer cells in the peripheral blood might be associated with her disease. Innate and adaptive immune cells are known to play significant roles in the host’s resistance to NTM infection and are associated with the exacerbation and progression of the disease (15, 16).

Patients receiving medication outside the hospital, without full supervision by a doctor, often do not undergo a further investigation into the causes of ineffective treatment, leading to prolonged misdiagnosis. Our patient discontinued her anti-NTM therapy, owing to physical intolerance, without consulting with a doctor. Therefore, we recommend that clinicians closely monitor patients with mycobacterial infections enrolled in treatment programs and gather clinical, imaging, and microbiological data. This approach is essential to evaluating treatment response and adherence. Timely identification of adverse drug reactions enhances the likelihood of successful treatment completion (17).

## 4 Conclusion

The NTM infections in lymph nodes can often be misdiagnosed in the absence of bacterial identification. In instances of prolonged etiological negativity, an experienced multidisciplinary team undertakes a comprehensive evaluation of all potential diagnoses. Clinicians should be alert to patients with long-term lymphocyte cell reduction. In such cases, employing rational and standardized sampling, along with a combination of various detection methods, is crucial for the effective diagnosis, management, and treatment of patients.

## Data Availability

The original contributions presented in the study are included in the article/supplementary material. Further inquiries can be directed to the corresponding author.
